# A Nobel-Winning Scientist: Aziz Sancar and the Impact of his Work on the Molecular Pathology of Neoplastic Diseases

**DOI:** 10.5146/tjpath.2020.01504

**Published:** 2021-05-15

**Authors:** Burcin Pehlivanoglu, Anil Aysal, Sibel Demir Kececi, Sumeyye Ekmekci, Ibrahim Halil Erdogdu, Onur Ertunc, Betul Gundogdu, Canan Kelten Talu, Yasemin Sahin, Muhammed Hasan Toper

**Affiliations:** Department of Molecular Pathology, Dokuz Eylul University, Graduate School of Health Sciences, Izmir, Turkey

**Keywords:** Aziz Sancar, Carcinogenesis, Molecular Pathology, Nobel prize, Neoplasia

## Abstract

Aziz Sancar, Nobel Prize winning Turkish scientist, made several discoveries which had a major impact on molecular sciences, particularly disciplines that focus on carcinogenesis and cancer treatment, including molecular pathology. Cloning the photolyase gene, which was the initial step of his work on DNA repair mechanisms, discovery of the “Maxicell” method, explanation of the mechanism of nucleotide excision repair and transcription-coupled repair, discovery of “molecular matchmakers”, and mapping human excision repair genes at single nucleotide resolution constitute his major research topics. Moreover, Sancar discovered the *cryptochromes*, the clock genes in humans, in 1998, and this discovery led to substantial progress in the understanding of the circadian clock and the introduction of the concept of “chrono-chemoterapy” for more effective therapy in cancer patients. This review focuses on Aziz Sancar’s scientific studies and their reflections on molecular pathology of neoplastic diseases. While providing a new perspective for researchers working in the field of pathology and molecular pathology, this review is also an evidence of how basic sciences and clinical sciences complete each other.

## INTRODUCTION

Henrich Rohrer, Nobel Prize winning physicist, once said that science requires constantly walking a tightrope between faithful belief and impulse to question, between common knowledge and creativity, between the defense of old territory and the decision to leave established grounds, between bias and impartiality, between expertise and fresh minds, between ambition and passion, between arrogance and self-confident conviction – in short, between human weakness and scientific standards, between today and tomorrow (1).

Scientists/researchers who have the patience to walk this tightrope are more likely to make discoveries that change the world, and one of them is Aziz Sancar, who was awarded the Nobel Prize in Chemistry with Tomas Lindahl and Paul Modrich in 2015 for their conceptual studies on DNA repair (2). Aziz Sancar was born in Savur, a small town in Southeast Turkey, in 1946. His fascination with biochemistry started in his second year of medical school when he learned about the DNA double helix for the first time (3). During his medical education at the Istanbul University Faculty of Medicine, he had the chance to work with excellent researchers including Dr. Mutahhar Yenson and Dr. Muzaffer Aksoy (4). After practicing in Mardin for about 2 years as a physician, he won a NATO fellowship and attended a PhD program at Johns Hopkins University in 1971. However, life had other plans for Sancar, and he left Johns Hopkins and returned to Turkey in June 1972. After a short break, he went to the United States in 1973, and received his PhD in 1977 at the University of Texas at Dallas (3,4). His research journey officially began while he was a PhD student. The road to success was rocky but in the end, he won the Nobel Prize, one of the most prestigious awards in the world.

Sancar’s discoveries (5–13) (Table 1) had a major impact on several scientific disciplines, particularly disciplines that focus on carcinogenesis and cancer treatment. As molecular pathology, a relatively new discipline that incorporates the morphology and molecular alterations of diseases, is among those disciplines, here we discussed the impact of Aziz Sancar’s studies on the molecular pathology of neoplastic diseases.

## FIRST SUCCESS and the LONGEST JOURNEY: *CLONING the PHOTOLYASE GENE*

The discovery of the photolyase enzyme, known as SANCAR’s enzyme in the literature, has taken 40 years of Sancar’s scientific life, and is very important since it contains the first information leading to the understanding of DNA damage repair in humans. The photolyase enzyme was first described in 1958 by Sancar’s mentor Dr. Rupert by observing the invigorating effect of blue light on bacteria (14). Dr. Rupert (14) showed that ultraviolet (UV) light can kill bacteria by damaging its DNA, and also revealed the presence of an enzyme that repairs DNA damage in visible light using blue light energy. UV was able to damage bacterial DNA by converting two adjacent pyrimidines, including thymines, into a CPD (cyclobutene pyrimidine dimer), while photolyase enzyme was helping to convert the abnormal thymine dimer into two normal thymine molecules using blue light energy (15–17)*. *However, Rupert and his team could not purify the enzyme due to the low level of enzyme content in bacteria, and therefore could not demonstrate how the enzyme converted sunlight into chemical energy, in other words how it repaired DNA damage caused by UV exposure.

Sancar has achieved great success by exploring the working principles and mechanism of photolyase and has pioneered the understanding of DNA repair mechanisms. After joining Dr. Rupert and his team, Sancar primarily aimed to clone the *photolyase* gene and obtain the enzyme in pure form. At this point, he first showed that the photo-activated photolyase can be transferred through the plasmids in the cell and that the plasmids can help in the replication process of this enzyme (18)*.* Subsequently, using recombinant DNA (rDNA) technology, which was newly revived at that time, he produced a mutant *E. coli *clone that did not have the photolyase enzyme. Using this mutant form, he enabled *E. coli* that could not repair its DNA to use blue light, thanks to the photolyase enzyme carried by the plasmid. In other words, his team demonstrated that they could treat the mutant *E. coli* by placing the photolyase gene in the normal *E. coli* chromosome into the plasmid (5,19)*. *In the meantime, they were able to replicate *photolyase* gene many times using the plasmid and succeeded in obtaining abundant (100-fold amplification) amounts of this enzyme (5)*. *Obtaining the enzyme easily and in abundance allowed them to investigate the repair mechanism of DNA damage by this enzyme using blue light. Therefore, in their subsequent studies, Sancar and colleagues (20–35) analyzed enzyme content and identified light-absorbing components and the working dynamics and revealed the 3D structure of the enzyme by crystallizing photolyases (36).

Sancar continues to work on the photolyase enzyme, which covers the longest period of his scientific journey, and he has also searched for its equivalent in human beings. In fact, the photolyase enzyme is very important in terms of introducing the DNA repair mechanism as a discipline from the first years it was described. Could the presence of this enzyme in bacteria but not in humans be a limitation for humanity? While there is no clear answer for this question yet, some researchers have been studying the possible use of photolyase in mRNA-based gene therapy in humans (37,38), and promising therapeutic agents containing photolyase have been reported to be used in the treatment of lesions triggered by UV rays (39,40). These agents have been combined with thermostimulation in another study (41)*. *However, the efficacy of these topical agents containing photolyase remains controversial and further investigation is needed to explore whether these agents are truly beneficial or not.

## DISCOVERY of the *“MAXICELL”* METHOD

Maxicell is a method developed by Aziz Sancar and colleagues (6) to identify the proteins encoded by the bacterial plasmid. This method may be used to produce any protein, and thus, has taken its place in biochemistry and molecular biology practice as a viable genetic engineering and rDNA technology method. The method uses a mutant, non-photo reactivable *E. coli* strain that cannot repair DNA under experimental light. Following irradiation, in about 6 hours, 80% of the bacterial chromosomal DNA breaks down and the irradiated mutant bacteria can no longer encode genes and therefore synthesize proteins. On the contrary, plasmids in the irradiated bacteria escape DNA damage due to their small size, and continue transcription and protein synthesis, and these gene products can be monitored using radioactive labeling (6). Although the irradiation limit that the plasmids can escape from is not known precisely, the maxicell method has been used up to 10x106 plasmid size (42).

While Sancar used this method especially in his studies on photolyase (5,43–45) and DNA repair by endo/exonucleases (46–48), many other researchers adopted this method, mainly in their studies that focused on viral and/or bacterial antigens (49–54). More importantly, the maxicell method has been used to produce some hormones (55,56) and monoclonal antibodies (57–59), i.e., molecules that can be both used for diagnostic and treatment purposes. The maxicell method has also facilitated the purification of recombinant gene products and production of DNA fragments (duplicates) in a fast and easy way, representing an initial step for nucleic acid amplification methods such as polymerase chain reaction (PCR) and other quantitative techniques. Currently, PCR is among the most frequently used techniques in molecular pathology practice.

## EXPLANATION of the MECHANISM of NUCLEOTIDE EXCISION REPAIR in *E. COLI* and HUMANS

DNA damage compromises the functional integrity of DNA (60) and occurs through multi-faceted mechanisms (60–64). When it occurs, the cells can either repair the damage, or stop the progression of the cell cycle, or induce apoptosis (65).

**Table 1:** Overview of Sancar’s greatest scientific discoveries.

**Discovery**	**Highlights**
***Cloning the photolyase gene***	· Sancar’s mentor, Dr. Claud S. Rupert, (14) discovered photolyase in 1958 (“the beginning of the scientific field of DNA repair” as referred to by Sancar himself in his Nobel lecture (65)). · Almost 20 years after this discovery, Sancar and Rupert (5) succeeded in cloning the photolyase gene, and this was the initial step of Sancar’s work on DNA repair mechanisms.
***Discovery of the “Maxicell” method***	· The maxicell method, discovered by Sancar (6), is used to identify plasmid-encoded proteins that use a mutant strain of *E. coli* that is defective in repairing DNA damage.
***Explanation of the mechanism of nucleotide excision repair in E. coli and humans***	· While cloning the excision repair genes *uvrA*, *uvrB*, and *uvrC*, Sancar and Rupp (7) found that UvrABC nuclease made dual incisions and named the enzyme “ABC excinuclease”. · Afterwards, Sancar and colleagues (8) discovered that dual incisions made during nucleotide excision repair in humans were different than in *E. coli*.
***Transcription-coupled repair:*** ***“Yunus Emre Opus”***	· Sancar and Selby (10) identified a factor that recognized and removed RNA polymerase from the damaged site while inducing the accumulation of the excision nuclease at the damage: TRCF (Transcription-Repair Coupling Factor). · Sancar describes this paper as his most aesthetically pleasing work, specifically as his “Yunus Emre Opus” (65).
***Discovery of “molecular matchmakers”***	· While studying DNA repair mechanisms, Sancar and Hearst discovered a class of proteins, “molecular matchmakers”, that promotes formation of a stable DNA-protein complex (9).
***Excision repair map of the human genome at single nucleotide resolution: “Piri Reis map”***	· Sancar and colleagues (11) mapped the sites of repair across the entire human genome, using XR sequencing and at single nucleotide resolution. · Sancar refers to this map as his Piri Reis map, while he describes it as the most satisfying accomplishment in his lab in the 2000s (4,65).
***Cryptochrome and the Circadian clock***	· In 1996, after reading an article about the circadian clock and jetlag in a flight magazine, Sancar began his studies on the circadian clock (4), showing that cryptochromes (named by Sancar himself) affect the clock (12,13), leading to several subsequent studies.

**Figure 1:**
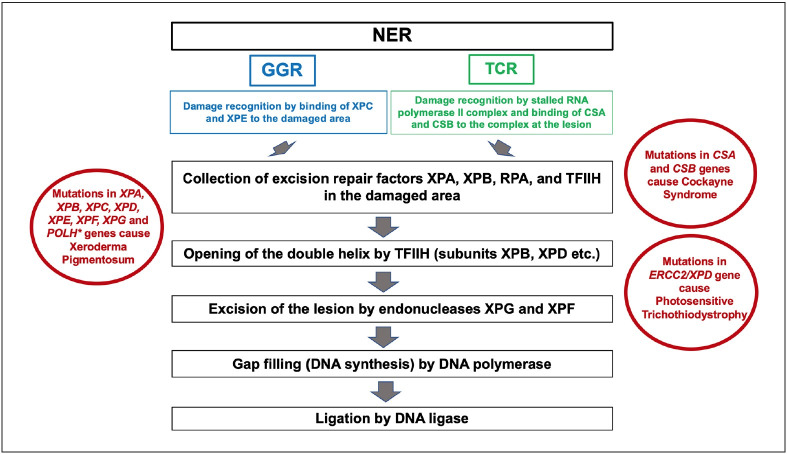
Basic steps of nucleotide excision repair (NER). While global genome repair (GGR) recognizes and repairs damages in the entire genome, transcription-coupled repair (TCR) operates on transcribed regions only (60,83,151). **TFIIH:** Transcription factor II Human. *POLH gene encodes DNA polymerase eta.

Nucleotide excision repair (NER) is a multi-component, multi-stage enzymatic system which includes recognition and elimination of a wide range of DNA damage (60–64). NER has been described in detail by Sancar et al. in prokaryotes and eukaryotes (60,61,65–67). All free-living organisms have excision repair genes (60,66). The damage is removed as a 12-13 nucleotide-long oligomer in prokaryotes and a 24-32 nucleotide-long oligomer in eukaryotes (60,63,68). NER starts with the recognition of the DNA damage, and then an oligomer, which has been formed by dual incision of the damaged area, is excised and released, and finally, the gap is filled by repair synthesis and ligation (60,67). These steps can be affected by many *in vivo* factors such as transcription, DNA replication, epigenetic modifications and/or binding of regulatory proteins to DNA (69). Six proteins (XPA, RPA, XPC, TFIIH, XPG and XPF-ERCC1 complex) are involved in excision repair in humans (60,61,68,70–72) (Figure 1). XPC, XPA and RPA are responsible for damage recognition, TFIIH (“Transcription factor II Human”) plays a role in DNA unwinding, and XPG and XPF-ERCC1 complex are responsible for 3’ and 5’ incisions (60).

Repair of UVB-induced pyrimidine dimers is important for the prevention of skin cancer development by NER mechanisms (64). Loss of DNA repair capacity due to NER deficiency causes genomic instability which is a carcinogenic feature (73). NER has been the focus of interest in various studies, both for understanding the pathogenesis and for the discovery of targeted therapies. Defective NER has been associated with three rare autosomal recessive hereditary diseases: xeroderma pigmentosum (XP), Cockayne syndrome (CS), and photosensitive trichothiodystrophy (TTD) (64) (Figure 1).

Among these three syndromes, pathologists often encounter XP while signing out skin resections. In XP, a defect in NER greatly increases the lethality and mutagenicity of the DNA-damaging agents and it is characterized by deficient nucleotide excision repair, extreme sensitivity to sunlight, and early onset skin cancer in humans (60,64,65,69). The risk of sunlight-induced skin cancers is greatly increased in XP patients, compared to the normal population (65,74). This finding is not surprising considering that excision repair in humans is the only known mechanism for eliminating UV-induced lesions (60).

NER deficiency has been demonstrated to be associated with non-skin cancer as well. In a study of breast cancer patients, mRNA expression levels of the NER genes have been shown to decrease in representative tumor samples compared to normal tissue samples by microarray analysis and these results have partially been confirmed at the protein level (73). Hence, the authors have suggested that NER deficiency may contribute to the development of sporadic breast cancer and that early-stage breast cancer may be sensitive to genotoxic chemotherapeutic agents such as cisplatin, the damage of which is eliminated by NER (73). Lu et al. (75) have reported that the polymorphisms of nucleotide excision repair genes *ERCC1 rs11615* and *ERCC5(XPG) rs17655 *are related to increased risk of laryngeal cancer but the biological effect of these polymorphisms is uncertain. Impaired NER has also been suggested to contribute to the development of head and neck squamous cell carcinomas (76). Defects in NER and base excision repair (BER), one of the main mechanisms of defense against oxidative DNA damage, have been reported to play a role in susceptibility to differentiated thyroid carcinoma (77). However, this effect appears to be due to some polymorphic genes with a weak overall effect (77).

Nevertheless, defective NER mechanisms do not contribute to the pathogenesis of some cancer types. For example, a minor relationship has been found between colorectal cancer and *XRCC3 *(a recombination repair gene) polymorphism, but the significance of this finding remains to be explored and NER genes are not considered to be major players in colorectal carcinogenesis (74). In another study, Gaddameedhi et al. (71) have found that melanoma cells retain their capacity for NER, and suggested that NER loss probably does not contribute much to the progression of melanoma.

In a study that measured the repair rates of UV-induced DNA damage during the differentiation of human embryonal carcinoma cells to neurons and muscle cells, NER capacity increased with the cellular differentiation level (62). Current standard anticancer therapies limit tumor growth by decreasing tumor proliferation or vascularization, but they have limited effects on cancer stem cells (78). A better understanding of the biological effect of potential NER inhibitors should facilitate the development of optimal synthetic killer combinations (78). This approach is promising not only for the individualized treatment of cancer patients with NER deficiency syndrome, but also in the treatment of patients with cancer in general (78).

## TRANSCRIPTION-COUPLED REPAIR: *“YUNUS EMRE OPUS”*

NER works more effectively in the transcribed regions of the genome. At this point, the “transcription coupled repair mechanism” comes into play. This mechanism, which is activated when RNA polymerase encounters DNA damage, does not allow the transcription to continue before the damage is repaired, provides faster repair of the damage, and reduces the harmful effects of transcription pause in the cell (79). The mechanism of this priority repair system, which is selective to the transcribed chain of the gene, had not been illuminated for a long time. In 1985, photodimers formed in the transcribed genes in various mammals and bacteria have been shown to be removed 5-10 times faster than the non-transcribed regions in the chromosome (80–82). Aziz Sancar and Christopher Selby (83) demonstrated that the progression of the RNA polymerase was stopped by DNA damage and caused the formation of a metastable RNA polymerase elongation complex at the damaged site, but this complex inhibited the repair rather than accelerating it in their study designed with damaged DNA, purified RNA polymerase and the UvrA, UvrB and UvrC proteins in *E. coli*. Consequently, they purified a protein in the form of translocase, which they named as “transcription-repair coupling factor (TRCF)”, which recognizes the RNA polymerase complex, separates it from the damaged area, and enables UvrA to come to the damaged area. Thus, they succeeded in enlightening the mechanism by finding the missing factor in the system. They also discovered that this protein was encoded by the *MFD* (mutation frequency decline) gene, which was known to prevent UV-induced DNA damage (10,84–87).

With this discovery, studies aiming to explain the mechanism of TCR in humans by various researchers including Aziz Sancar revealed that the damage recognition step was carried out by stopped RNA polymerase II complex, differently from global repair, and that this complex collected CSB translocase and the core excision repair factors except XPC to the damaged area and the next steps continued the same as in global repair (11,60,88–91) (Figure 1).

The reflections of the studies on this topic to the clinical medicine and molecular pathology can be grouped in three categories: 1) effects on cellular aging, carcinogenesis, apoptosis; 2) effects on cisplatin susceptibility and resistance, and 3) the potential use in targeted therapy.

There is evidence that the RNA polymerase II complex activates *P*53, initiates apoptosis, and acts as the primary sensor in all DNA damage response reactions (92). Mutations that inactivate TCR are known to cause Cockayne Syndrome in humans (11,93). Mutations occurring in the *CSB/ERCC2* gene encoding TRCF and some mutations that disrupt repair function in *XP* genes cause Cockayne Syndrome (85) (Figure 1). Damaged TCR system causes the DNA damage to stop transcription, resulting in impaired cell function, premature aging, and cell death. The effects of TCR damage differ from tissue to tissue depending on factors such as tissue metabolism, activity of antioxidant systems, and other repair systems. This explains the clinical picture seen in Cockayne Syndrome, which is the most well-known TCR-associated disorder, and is characterized by cellular aging in tissues consisting of non-proliferating or slow proliferating cells such as Schwann cells and neurons, resulting in progressive neurodevelopmental disorders. The delicate balance between global repair disorders that cause cancer development and TCR disorders that cause premature aging is critical in preventing both cancer and premature aging. The TCR system, which is still intact in global repair disorder, ensures cell survival and delayed cellular aging, but results in accumulation of DNA damage in the non-transcribed genes and the non-transcribed chain of active genes, thereby causing mutagenesis and an increased risk of cancer. On the other hand, cells that cannot be repaired by the TCR system die due to transcriptional stress providing a strong protection against cancer (94). From this point of view, the use of TCR-inhibiting or -blocking agents is promising as an adjunctive treatment approach to chemotherapy. In this way, it is thought that tumor cells will become more sensitive to the lethal effects of chemotherapy and at the same time, surviving tumor cells will not carry the treatment-induced mutations (95).

Resistance to chemotherapy is an important problem in cancer treatment. The sensitivity to cisplatin-based treatments, which are commonly used in cancer management, has been reported to be increased in cells with TCR damage. It has been stated that detection of TCR disorders in cancer cells may be helpful in predicting resistance to cisplatin treatment (96). Cisplatin resistance is thought to be related to increased DNA repair capacity, *P53* mutations, or loss of DNA mismatch repair capacity. There is evidence that reduced TCR capacity increases the susceptibility of tumor cells to apoptosis induced by cisplatin even in cell lines with *p53* mutation and DNA mismatch defects, and that the TCR system may be a potential target in overcoming cisplatin resistance in cancer treatment (97). For example, in experimental studies in chronic lymphocytic leukemia (CLL), TCR inhibition, in addition to the treatment regimen, has been shown to induce cell death regardless of the previous treatment and to have synergistic effects with the treatment regimen, revealing that it is a mechanism that can be used in refractory disease to re-sensitize CLL cells (98,99).

## DISCOVERY of “MOLECULAR MATCHMAKERS”

Sancar and Hearst discovered “molecular matchmakers” while studying DNA repair mechanisms. They defined the “molecular matchmakers” as a class of proteins that make conformational (structural) changes in at least one of the DNA-binding protein pairs to increase the formation of the DNA-protein complex (9). Structural changes facilitate and stabilize the protein-DNA complex formation. The degree of change enhances the specificity and stability of the complex (100). The molecular matchmaker is a protein that combines two compatible but also solitary macromolecules with an ATP-dependent reaction, promotes their fusion, and then leaves the new complex to continue its processes (9,101). Molecular-matchmakers are not only involved in DNA-protein binding but also in other molecular interactions, such as RNA-protein, RNA-DNA and macromolecule-small ligands (101).

Protein interactions are essential in all stages of homeostasis. Therefore, elaboration of these interactions provides unique opportunities to understand the molecular basis of diseases, and to develop better diagnostic and treatment strategies. It is crucial to characterize biochemical, physical and functional aspects of protein interactions (102,103). Mutations that affect protein structure may cause impaired protein-DNA interactions, protein misfolding, new unwanted protein interactions, or pathogen-host protein interactions (9,102). For instance, mutations in *P53*’s DNA-binding domain impair its ability to bind to target DNA sequences, blocking several tumor suppressing mechanisms such as apoptosis, genetic stability etc. (102). It has already been demonstrated that misfolded and aggregated proteins may cause several diseases, either by disruption of specific binding abilities, formation of unwanted proteins, and/or over-accumulation of the impaired protein (104–106). Viruses that can integrate their genetic material to the host genome such as the human papilloma virus (HPV) and hepatitis B virus (HBV) can initiate and/or promote carcinogenesis via pathogen-host protein interactions (107,108).

Studies on protein interactions help to predict genotype-phenotype associations, and new diagnostic tools can be created from these associations. Identifying involved pathways serves as a key to discover new diagnostic and prognostic tools. The links of diseases and proteins help us find key areas as potential drug targets and provide information for drug design (102). For example, the heterogeneous nuclear ribonucleoprotein (hnRNP) A2/B1, which is an RNA matchmaker (109), has been shown to promote carcinogenesis, invasion and metastasis in pancreatic ductal adenocarcinoma, glioblastoma and lung cancer, but to play an inhibitory role in metastasis in breast cancer, making it a promising prognostic biomarker and a potential molecular therapeutic target for breast cancer (110).

## EXCISION REPAIR MAP of the HUMAN GENOME at SINGLE NUCLEOTIDE RESOLUTION: *"PIRI REIS MAP"*

Sancar and colleagues (11) have recently mapped the sites of repair across the entire human genome at single nucleotide resolution using XR (excision repair) sequencing. Sancar refers to this map as his “Piri Reis map” (4,65) showing a “new world of repair genes”, referring to Piri Reis, an Ottoman admiral and cartographer who drew one of the oldest maps of the New World. During excision repair, a single strand with 30 nucleotides that includes the lesion is removed from the DNA after dual incisions. XR-sequencing basically depends on capturing and sequencing this excised strand, allowing to create a genome-wide map of human excision repair at single-nucleotide resolution and provides valuable data about the effects of genomic position and chromatin status on DNA damage and repair (11). Using the same sequencing method, they then conducted other studies investigating the effects of anti-cancer drugs such as cisplatin on DNA damage and repair (111–113). For example, in a study aiming to understand DNA damage caused by cisplatin and its repair in the mouse liver by using XR-sequencing, Sancar and colleagues (111) showed that the repair of the transcribed strand is dominant in the first two days after cisplatin injection and then the repair of the non-transcribed strand becomes dominant, an information that may be useful for designing chemotherapy regimens. Another XR-sequencing-based study in colon cancer cell lines demonstrated up-regulation of membrane transport pathways in the oxaliplatin-resistant cells (113). Such studies using the XR sequencing method may provide an improvement in the way of illuminating the cellular response or resistance mechanisms to anti-cancer drugs.

## ROLE of the CIRCADIAN CLOCK in CARCINOGENESIS and CHRONOTHERAPY: A NEW APPROACH in CANCER TREATMENT, “CHRONOCHEMOTHERAPY”

The circadian rhythm (also called the “biological clock” (“circa” meaning “approximately”, “diem” meaning “day” in Latin) is a mechanism regulating physiological and metabolic events in living creatures in the form of daily endogenous rhythms over a 24-hour period. Although it demonstrates an endogenous oscillation, the circadian rhythm uses some signals received from the environment, such as light and nutrition, as determinants in the regulation of rhythms. The circadian clock is a universal regulatory system that creates daily rhythms in multiple physiological periods interfering with other regulatory systems and pathways in mammals in order to ensure the stabilization of homeostasis (114). It influences the physiological rhythm and metabolic events significantly (111).

In humans, the circadian clock has been determined to be formed under four gene controls called *CLOCK, BMAL1, Cryptochrome* and *Period* (114). The cryptochrome genes discovered by Sancar and Miyamoto (115,116) regulate the circadian clock in plants and animals. Sancar (12) named the cryptochromes (*CRY1 and CRY2)* based on their resemblance to the plant blue light photoreceptors which also had sequence similarity to photolyase. In this cycle, as the transcriptional activators, CLOCK and BMAL1 proteins bind to promoters of the *Cryptochrome* and *Period* genes to activate their transcription, followed by the inhibition of CLOCK-BMAL1-activated transcription (117). The CLOCK – BMAL1 complex has been shown to affect up to 10% of the entire transcript in the circadian cycle (118). Loss of several tumor suppressor mechanisms caused by an impaired circadian rhythm has been suggested to contribute to carcinogenesis (119–121). Deregulated expression of numerous circadian cycle proteins has been found to be associated with poor prognosis and aggressive behavior in several malignancies (122–127). It has also been shown that single nucleotide polymorphisms in the *CRY2 *and *CLOCK* genes increase the risk of breast (128) and colorectal cancer (129).

On the other hand, whether the disruption of the circadian clock is a risk for cancer remains unclear due to paucity of supporting data from well-controlled genetic studies on mice (114) and the presence of findings indicating otherwise (130), despite the supporting evidence from experimental studies (131,132). In the few limited epidemiological studies that investigated the relationship between circadian rhythm and cancer development, a higher risk of breast, prostate and colorectal carcinoma have been reported in people who work in the night shift (133–135). As of 2019, the International Agency for Research on Cancer (IARC) defines night work as “group 2A, probably carcinogenic to humans” based on the limited evidence on the subject (136). Hence, further investigation is required to fully elucidate the relationship between the circadian rhythm and carcinogenesis.

Nevertheless, the potential use of therapeutic agents to re-regulate the circadian rhythm to cure human diseases and to develop anti-cancer drugs targeting circadian clock genes and proteins are still hot research topics (137–139). Moreover, administration of chemotherapy based on the circadian rhythm (“chrono-chemotherapy”) has opened a new era. Dysregulation of the circadian clock can affect cancer susceptibility by regulating DNA damage repair mechanisms and apoptosis. The NER mechanism has also been demonstrated to be regulated by the circadian rhythm. Sancar et al. (140) have observed that NER increased gradually in the morning and reached its highest level in the evening, and that mice exposed to UVB radiation in the morning hours developed four times more invasive skin cancer compared to the evening hours. Subsequently, they investigated the most convenient time during the day for administration of cisplatin, a chemotherapeutic agent, in order to reduce cisplatin’s adverse effects as the damage caused by cisplatin on DNA is repaired by the NER system, and suggested that it can be administered when DNA repair in normal tissues is the highest during the day, i.e., in accordance with the circadian rhythm (111,141). Other researchers have also reported promising results for the use of chrono-chemotherapy (142,143). Although evidence on the efficiency of chrono-chemotherapy remains to be fully elucidated, Sancar and colleagues have suggested that further research may provide information on the efficacy and feasibility of timed administration of cisplatin (141), and their detailed research on this topic is ongoing.

## FUTURE REMARKS

Aziz Sancar’s studies had a remarkable impact on other disciplines, especially on cancer research. His discoveries on photolyase (144) and NER have already led to studies on carcinogenesis (74–76,145), and targeted therapy options are being investigated based on his work on NER (146,147), TCR (96,98), cryptochromes, and the circadian clock (114). The future of targeted therapy, at least in part, seems to be built around some of his studies.

Sancar is a living proof of the saying “Science knows no country, because knowledge belongs to humanity, and is the torch which illuminates the world.” (148). Who would have thought someone from a small town in Turkey would go all the way to the Nobel Prize? But then, life is all about surprises. And now, it is time for junior scientists/researchers to work on their take-home messages from Sancar’s studies. To date, only few pathologists have won the Nobel Prize (and in different categories). Some pathologists have claimed that pathologists could not receive the Nobel Prize, because morphology alone was not enough, and with the emergence of molecular pathology, this obstacle may be overcome (149). Regardless, there is a bigger reward that science promises to researchers: “The emotional thrill of being the first person in the history of the world to see or to understand something (150)”, and Sancar’s success is an inspiration for junior researchers.

## CONFLICT of INTEREST

The authors declare no conflict of interest.

## FUNDING

The authors received no specific funding.
